# Nrf2 Transcriptional Activity Governs Intestine Development

**DOI:** 10.3390/ijms23116175

**Published:** 2022-05-31

**Authors:** Aleksandra Kopacz, Damian Kloska, Dominika Klimczyk, Magdalena Kopec, Alicja Jozkowicz, Aleksandra Piechota-Polanczyk

**Affiliations:** Department of Medical Biotechnology, Faculty of Biochemistry, Biophysics and Biotechnology, Jagiellonian University, 30-387 Krakow, Poland; aleksandra.kopacz@doctoral.uj.edu.pl (A.K.); damian.kloska@uj.edu.pl (D.K.); d.klimczyk@student.uj.edu.pl (D.K.); magdalena1.kopec@student.uj.edu.pl (M.K.); alicja.jozkowicz@uj.edu.pl (A.J.)

**Keywords:** Nrf2, Nrf2 transcriptional activity, hindgut, intestine, gestation

## Abstract

Our recent findings indicate that Nrf2 transcriptional activity is essential in maintaining the proper large intestinal structure in adult mice. Here, we aimed to verify whether Nrf2-related intestine abnormalities stemmed from the early weaning or gestational periods. Therefore, we analyzed 4-day-old pups and embryos devoid of Nrf2 transcriptional activity (tKO) and their wild-type counterparts. We found significant changes in the intestinal structure of 4-day-old Nrf2 tKO pups including a longer colon, altered crypt distribution, and enlargement of the goblet cells with a markedly higher level of mucin 2. Tracing back the origin of these alterations, we observed that they appeared as early as day 14.5 of embryonic development, independently of sex. Importantly, in this period, we observed a significant increase in the Nrf2 level and a distinctive, untimely pattern of expression of the proliferation factor Ki67. At the latest stage of embryonic development, we detected a premature drop in the differentiation factor Notch1. We suspect that intestine abnormalities in mice lacking Nrf2 transcriptional activity stem from sex-independent disturbed intestinal cell proliferation and could be further exacerbated by altered differentiation. Summing up, we identified Nrf2 transcriptional activity as an important regulator of intestinal formation. It influences the hindgut cell proliferation and differentiation at different stages of embryonic development.

## 1. Introduction

The proper functioning of the gastrointestinal system requires intricate crosstalk between a plethora of different cell types, which interact and function in a strictly organized manner. Any derangement in the component activities, caused, for example, a dysfunctional resolution of inflammation is conducive to bowel diseases. Despite mounting research data and identification of the key factors governing proper intestinal homeostasis, we still lack an exhaustive description of the disease-causing factors [1,2]. Recent advances in the field have pointed out striking similarities between the fetal gut development and bowel disease transcriptional programs [3,4]. Therefore, addressing the origin of intestinal abnormalities and the identification of key players during embryonic intestinal growth may be of utmost importance for the understanding of the origin of many bowel diseases.

In mice, the primitive gastrointestinal tract (tube) is established around day E9.5. Following its growth, from day E14.5, the key milestones in proper hindgut formation occur including epithelial remodeling, transient emergence of villi, and the formation of crypts and stem cell niche. At these stages, colon homeostasis appears, ensuring a specialized type of cells. First, mucin-producing goblet cells emerge, followed by the appearance of hormone-secreting chromogenin A-positive enteroendocrine cells. The proper orchestration of these processes is ensured by elaborate reciprocation between the signaling pathways, mainly driven by Notch and Wnt. Importantly, these remain pivotal regulations of gut function during adult life [5,6]. Of note, our recent studies have pointed to another crucial factor in the maintenance of proper intestinal structure—the Nrf2 transcription factor [7].

Nrf2 (nuclear factor erythroid 2-related factor 2) is a stress-activated transcription factor governing cellular homeostasis. Through transcriptional activity, it regulates the expression of many antioxidative and detoxification enzymes, hampers the inflammatory response, and participates in a wide range of cellular processes. The interaction with the repressor Keap1 accounts for the fine-tuning of its activation. Several studies have emphasized the importance of the interplay and balance between Nrf2 and Keap1 in the maintenance of proper organismal function including proliferation and intestinal fitness (exhaustively reviewed in [8]). Of note, the impact of Nrf2 and oxidative stress has been widely attributed to the pathogenesis of inflammatory bowel disorders [9,10,11]. Moreover, the polymorphism in the Nrf2 promoter is interrelated with the onset of ulcerative colitis [12].

Given the pronounced Nrf2 mRNA level in the hindgut during gestation [13] as well as the significant association of Nrf2 with the master regulator of intestinal differentiation Notch1 [14,15] and with the regulation of cellular proliferation [16], we speculated that the Nrf2-related intestinal abnormalities would appear in the early gestation or early weaning period. To address this issue, we decided to assess the colon morphology at postnatal day 4 and, if rationale, at the earlier timepoints during embryonic development. We suppose that abrogation of the Nrf2 transcriptional activity alters cell proliferation and differentiation in the intestine, which accounts for the observed changes, therefore, we inspected the proliferation factor Ki67 and differentiation factor Notch1.

## 2. Results

### 2.1. Nrf2 tKO Pups Have a Longer Colon with Coexistent Microscopic Alterations

**Figure 1:**
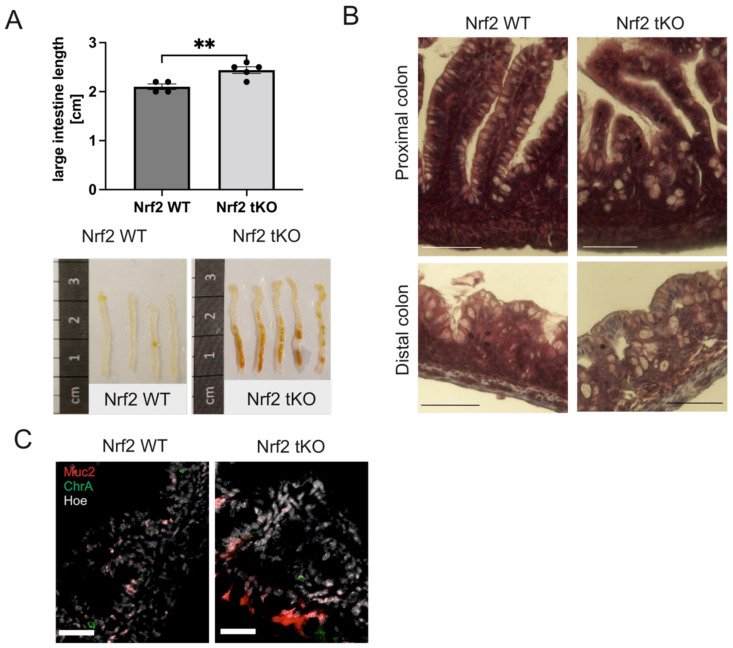
**Lack of transcriptional activity of Nrf2 changes colon morphology in 4-day-old pups.** (**A**) Macroscopic changes in the colon length isolated from 4-day-old mice with similar body weight; brown debris in the Nrf2 tKO are remnants of indigested food that was unmovable from the gut despite extensive flushing. (**B**) Hematoxylin and eosin staining of the proximal and distal colon showing the disruption of the colon crypts and enlargement of the goblet cells. (**C**) The presence of enteroendocrine (ChrA) and goblet (Muc2) cells in the colon. ChrA (green), Muc2 (red), and nucleus (gray). N = 3 for the Nrf2 WT and Nrf2 tKO mice. ** *p* < 0.01; Representative images. Muc2—mucin 2, ChrA—chromogranin A. Mean ± SEM. Student’s *t*-test. Magnification 400× for B, scale bar 30 µm and magnification 250× for C, scale bar 45 µm.

To address the origin of Nrf2-related colon alteration in older mice [7], we decided to inspect the colon from 4-day-old pups. We discovered that Nrf2 tKO pups had a significantly longer colon (Figure 1A), which was not caused by a higher mouse weight [16]. Peering closer into the large intestinal morphology, we observed a disruption of the colon crypts and an enlargement of the goblet cells in the proximal and distal colon (Figure 1B).

Given the presence of visible brownish residues of indigested food in the colon of the tKO pups (Figure 1A), which could result from the higher mucin content in those mice, we inspected the mucin-producing goblet cells and enteroendocrine cells. Interestingly, we noticed that mucin 2 (Muc2) was widely expressed in the epithelial layer of the Nrf2 tKO mice, whereas in the wild pups, the Muc2 level was markedly lower and was evenly spread within the epithelium and lamina propria. In contrast, the enteroendocrine chromogranin A (ChrA) containing cells were scarcely detectable in both genotypes (Figure 1C). To further verify whether these changes had already appeared during gestation or occurred only after birth, we further analyzed the intestinal development during gestation in the Nrf2 tKO and wild-type mice.

### 2.2. tKO Embryos Have Significantly Altered Crypt Distribution and Enlargement of Goblet Cells as Early as at Day 14.5 of Embryonic Development

**Figure 2:**
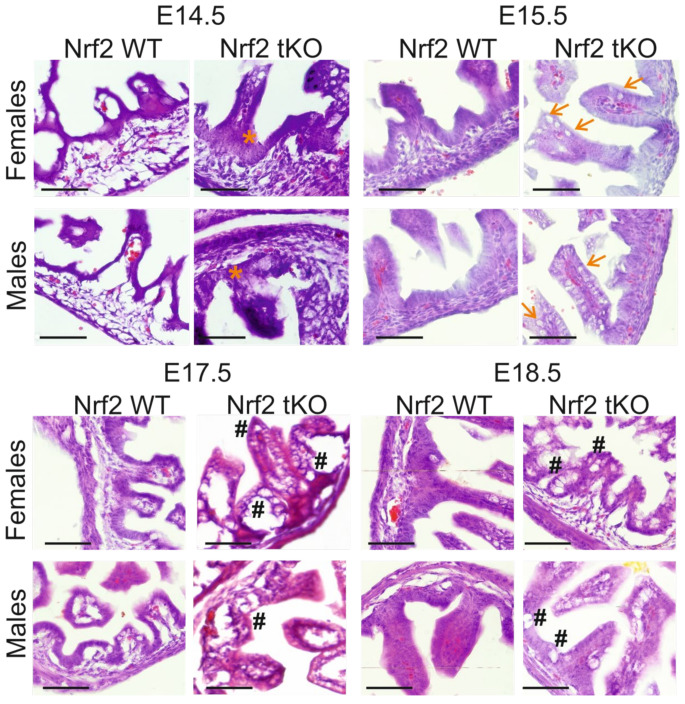
**Significant histological abnormalities in the hindgut of Nrf2 tKO embryos.** Hematoxylin and eosin staining of the intestine showed an enlargement of the epithelium in Nrf2 tKO fetuses at E14.5 (orange asterisk), earlier appearance of goblet cells at E15.5 (arrows), and further irregular organization and difference in the size of the goblet cells on days E17.5 and E18.5 (hash). N = 3–11 fetuses for the Nrf2 WT and Nrf2 tKO mice. Representative images, magnification 400×, scale bar 30 µm.

Significant intestinal morphogenesis is underway around day 14.5 of embryonic development [6], thus it became the starting point for our analyses. Microscopic evaluation of the intestinal structure during gestation showed that, contrary to the wild-type, Nrf2 tKO embryos had visible microscopic alterations in the intestines, manifested by the enlargement of the epithelium in Nrf2 tKO (E14.5; orange asterisk) and the earlier appearance of structures that resembled goblet cells (E15.5; arrows). These changes persisted during further stages of embryonic development and were sex independent. On day E17.5, tKO embryos displayed a more advanced irregular organization and an increase in the size of the goblet cell-like structures. These changes were further present on day E18.5 (Figure 2, hash).

**Figure 3:**
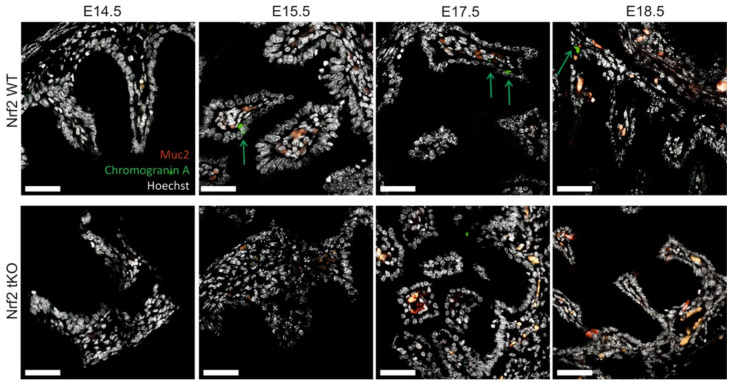
The Nrf2 transcriptional activity influences epithelial cell differentiation and the presence of enteroendocrine (ChrA) and goblet (Muc2) cells. ChrA (green), Muc2 (red), and nucleus (gray) expression in the female and male fetuses at selected gestation days; no sex-dependent changes. Green arrows—chromogranin A containing cells. N = 3–11 fetuses for the Nrf2 WT and Nrf2 tKO mice. Representative images, magnification 400×, scale bar 30 µm.

To further verify the types of cells that are present in the hindgut, we analyzed the expression of Muc2 and ChrA. Selected cell types were present in the Nrf2 tKO mice not sooner than at day E15.5 compared to the Nrf2 WT mice where single Muc2-containing goblet cells were visible at day E14.5. The number of Muc2-positive cells was similar between genotypes at E18.5 (Figure 3). The ChrA containing enteroendocrine cells were scarcely present in the Nrf2 tKO mice, while in the wild-type embryos, the ChrA positive cells were noticed at day E15.5 (Figure 3, green arrows).

### 2.3. Nrf2 Level Is Increased in the Hindgut during Gestation

**Figure 4:**
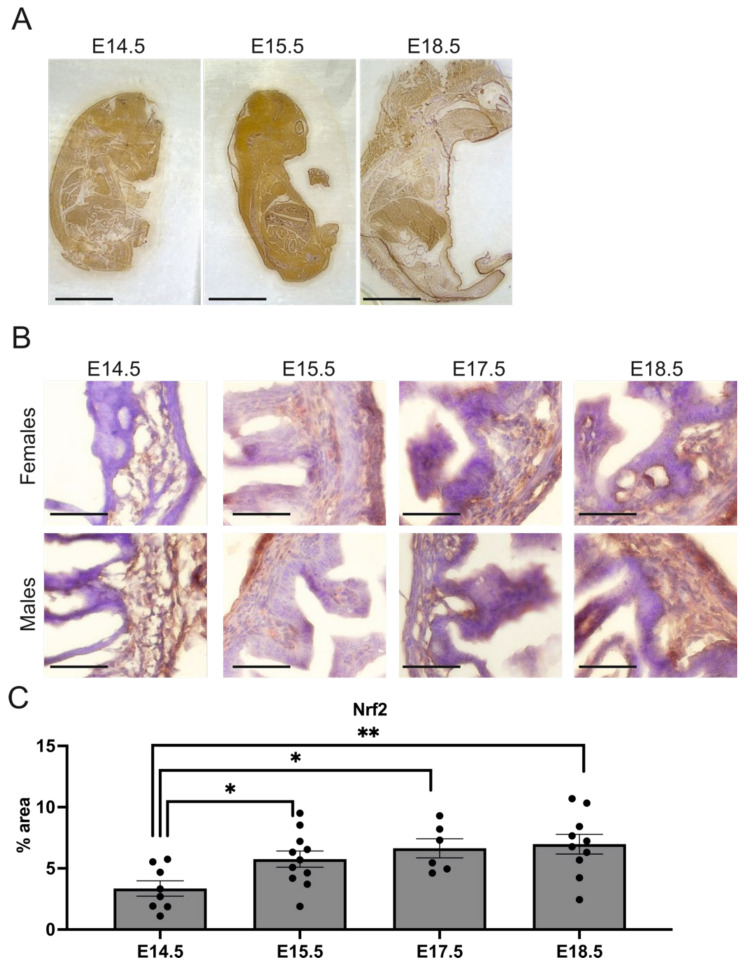
The Nrf2 expression changes in the embryo and hindgut during gestation. (**A**) The Nrf2 protein expression in embryos in the selected embryonic development days. (**B**) Nrf2 expression in female and male fetuses at selected gestation days. (**C**) Quantification of Nrf2 in the hindgut in Nrf2 WT fetuses; N = 6–11 fetuses of the Nrf2 WT mice. Mean ± SEM. One-way ANOVA. * *p* < 0.05, ** *p* < 0.01. Representative images, magnification 4×, scale bar 10 mm for A; magnification 400×, scale bar 30 µm for B.

The considerable changes in the intestinal structure upon Nrf2 transcriptional inhibition imply the importance of Nrf2, especially between days E14.5 and E15.5, when the most prominent changes occur (Figure 2). In accordance, we observed that Nrf2 expression changed during gestation in whole embryos (Figure 4A), peaking at day E15.5. A scrutinized inspection of the embryonic hindgut pointed to a gradual increase in Nrf2 during development, which was around 2-fold higher at E18.5 compared to E14.5 in the wild-type mice, especially in the lamina propria (*p* < 0.01) (Figure 4B). Importantly, as in the histological analyses, the changes were sex independent.

### 2.4. Nrf2 Transcriptional Activity May Influence the Proliferation and Differentiation at Different Stages of Embryonic Development

**Figure 5:**
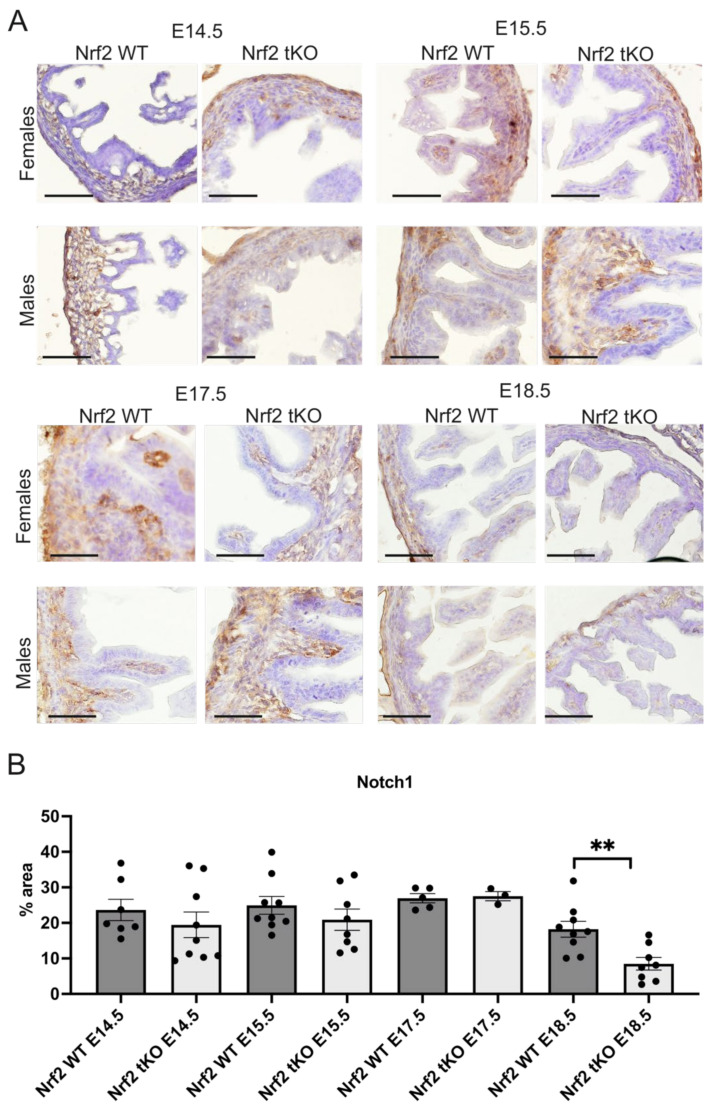
Notch1 is reduced in the Nrf2 tKO embryos at the latest stages of development. (**A**) Notch1 expression in the female and male fetuses at selected gestation days. (**B**) Quantification of Notch1 in the hindgut of the Nrf2 WT and Nrf2 tKO fetuses. N = 3–11 fetuses for the Nrf2 WT and Nrf2 tKO mice. Mean ± SEM. Two-way ANOVA. ** *p* < 0.01. Representative images, magnification 200×, scale bar 50 µm.

The abnormal regulation of Notch during development may alter epithelial proliferation and differentiation in the intestines [5,6] whereas the Notch protein level was comparable until day E17.5, when there was a significant reduction in the Nrf2 tKO mice at day E18.5 compared to the Nrf2 WT embryos (*p* < 0.01, Figure 5). There was no sex-related difference in all of the inspected parameters.

**Figure 6:**
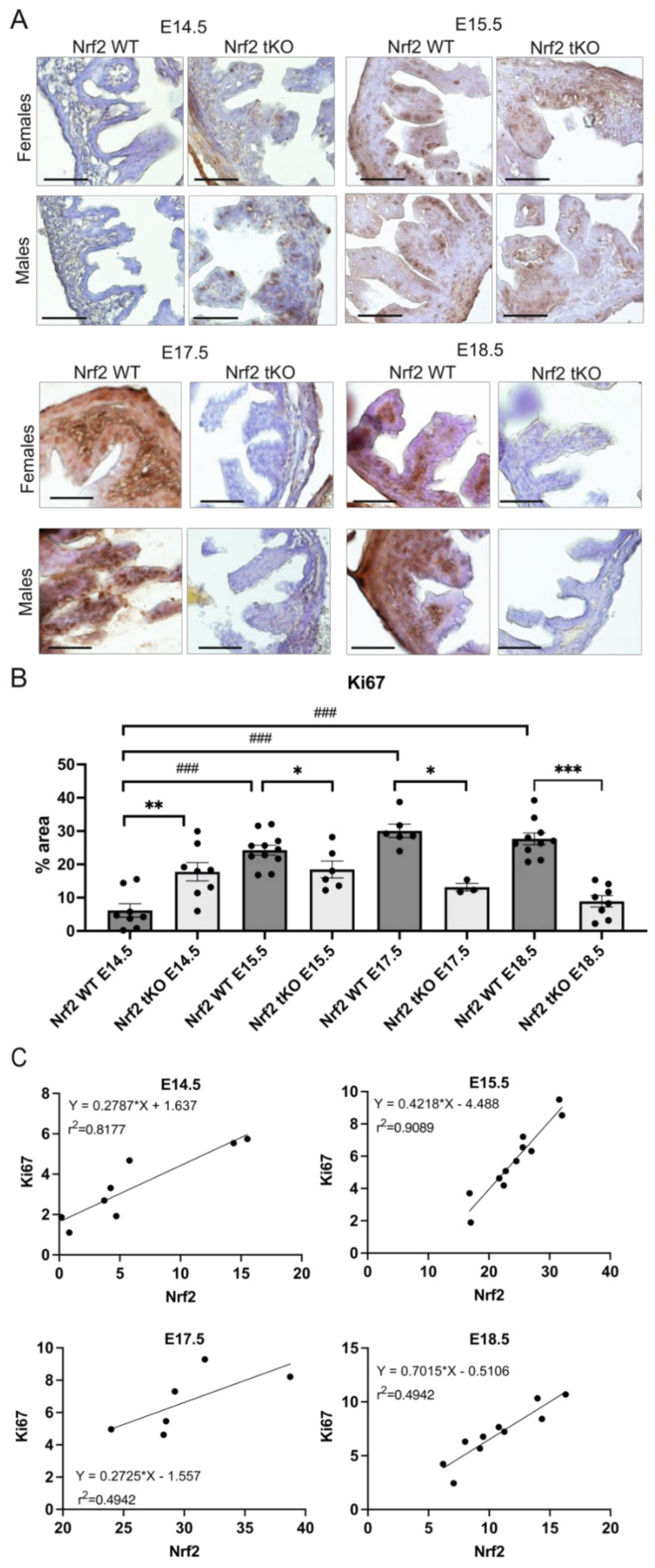
The differential pattern of Ki67 expression under Nrf2 inhibition. (**A**) Ki67 expression in the female and male fetuses at selected gestation days. (**B**) Quantification of the Ki67 protein level changes in the hindgut in the Nrf2 WT and Nrf2 tKO fetuses. (**C**) Correlation between the mean changes in Nrf2 and Ki67 in the analyzed gestation days in the WT embryos. N = 3–11 fetuses in the Nrf2 WT and Nrf2 tKO mice. Mean ± SEM. Two-way ANOVA. * *p* < 0.05, ** *p* < 0.01, *** *p* < 0.001, ^###^ *p* < 0.001. Representative images, magnification 400×, scale bar 30 µm.

Moreover, as we observed more numerous epithelial cells in the hindgut of the Nrf2 tKO mice during gestation (Figure 2), we further wanted to verify the expression of the proliferation marker Ki67. In the Nrf2 WT embryos, there was a gradual and significant elevation in Ki67. The expression of Ki67 was 3-fold higher at day E15.5 compared to E14.5 and peaked at E17.5. In contrast, in the Nrf2 tKO embryos, Ki67 topped at day E14.5, when it was twice as high in comparison to the wild-type counterparts. During the following days, the Ki67 level notably decreased and was substantially lower in the Nrf2 tKO (Figure 6A,B). Our further analysis indicated a strong positive correlation between Nrf2 and Ki67 in the wild-type mice in all of the analyzed gestation days, suggesting a possible regulation between Ki67 and Nrf2 transcriptional activity during the hindgut’s development (*p* < 0.001 for E14.5, *p* < 0.05 for E15.5, *p* < 0.01 for E17.5, and *p* < 0.0001 for E18.5) (Figure 6C).

## 3. Discussion

Here, we present new insights into how the transcriptional activity of Nrf2 impacts on the intestinal development and differentiation of enterocytes. Nrf2 transcriptional abrogation led to pronounced colon elongation, altered crypt distribution, enlargement of the goblet cells, and markedly higher levels of mucin. These alterations stem from sex-independent, disturbed intestinal maturation during gestation, plausibly associated with a distinctive regulation of proliferation and earlier Notch1 reduction.

This study was performed on mice solely lacking Nrf2 transcriptional activity. The N-terminal part of Nrf2 protein, responsible for the interaction with Keap1, was still present [16]. Of note, the debilitation of Nrf2 transcriptional activity did not trigger any apparent embryonic abnormalities. The homozygous knockout mice follow a normal Mendelian inheritance pattern, they are fertile, and do not manifest any easily recognizable disease phenotype [13,17]. Under basal conditions, they are indistinguishable from other littermates of different genotypes, apart from lower birth weight, when bred from heterozygotes [16,18].

Whereas the impact of Nrf2 on intestinal homeostasis has become better recognized [10], its role in gastrointestinal tract development remains elusive. Over 25 years ago, Chen et al. showed that Nrf2 mRNA levels are high in the luminal side of the intestines during murine gestation. Moreover, they reported significant variations in the Nrf2 mRNA levels between the organs and gestational days [13]. Here, we confirm these findings at the protein level. For instance, we noticed a continuous increase in the Nrf2 levels in the hindgut between E14.5 to E18.5 while in other tissues (e.g., the lungs or heart), the Nrf2 levels decreased. Therefore, these results point out that Nrf2 may play a pivotal role in intestine development during these days.

Importantly, in the same paper [13], Chen et al. showed that for all of the inspected organs, the mRNA level for the Nrf2-LacZ fusion protein (lacking transcriptional activity, but binding Keap1) was the highest in the intestines. This raises the essential question regarding the origin of intestine alterations in the Nrf2 tKO mice. In particular, it should be kept in mind that the phenotype of Nrf2 tKO mice is largely dependent on the balance between Nrf2 and Keap1 (summarized in [8]). Supportively, the Keap1 deficiency in mice is lethal at the weaning age due to the abnormal formation of the gastrointestinal tract [19] and a significant deregulation in enterocyte proliferation [15]. Therefore, the Keap1-related mechanisms governing hindgut formation cannot be excluded.

The main inspiration for this study came from the observations made in our previous research, where we investigated the role of Nrf2 transcriptional activity in response to a single-dose anesthetic in mice. We noticed in 6-month-old vehicle-treated Nrf2 tKO control mice significant macroscopic and microscopic changes in the gastrointestinal system including colon shortening [7]. Whereas the histological features remained similar, regardless of the age of the mice, here we report a significantly longer colon in 4-day-old Nrf2 tKO pups. We suspect that the source of this difference was either the impact of the vehicle (2.5% iso-amyl alcohol) used in the previous study or the age-dependent shortening of the colon.

In this paper, we aimed to extend our initial observations and histological analyses by more detailed verification of the type of cells affected in the developing intestine. Recently, it has been shown that the level of mucin 2 (Muc2) is gradually increased in the intestine during gestation, starting from around day E14.5 [3]. This is in line with our findings in the wild-type embryos. In the Nrf2 tKO embryos, although Muc2 appeared later (at E15.5), it reached a similar level at E18.5, and was markedly higher in 4-day-old pups. Interestingly, despite the higher mucin content, there was unmovable brownish “debris” in the intestine of the Nrf2 tKO pups. We presumed that it could be related to a distinctive type of mucus or its abnormal secretion [20]. Muc2 consists of two layers in the intestine: a thinner inner “firm” mucus layer, which is considered devoid of bacteria, and a thicker outer “loose” mucus layer, in which anaerobic microbiota may reside [21]. The brownish debris may reflect the presence of “firm” mucus, which hosts little bacteria. Therefore, as Muc2 alone or coupled with bacterial antigens can deliver immunoregulatory signals to both the underlying dendritic cells and intestinal epithelial cells [22], we suppose that this function may be debilitated in our Nrf2 tKO mice.

Our attempts to identify a trigger behind the histological alterations observed in Nrf2 tKO embryos pointed out the distinctive expression pattern of the proliferation factor Ki67. Recently, Fazilaty et al. reported that the level of proliferation markers such as Cdk1 and Ki67 changed in the intestine between E14.5 and E18.5. It was high at E14.5–15.5 and dropped at E18.5, which may reflect a shift toward a more differentiated cellular composition at later stages [3]. In the WT embryos, we observed a marked 3-fold increase in the Ki67 protein level between E14.5 and E15.5, then the level remained stable. We did not observe the aforementioned drop at day E18.5. This discrepancy may stem from the fact that in the referred research [3], mRNA was analyzed, whereas, here, we inspected the protein level. Importantly, Ki67 has a half-time of 36 h under newly induced quiescence [23].

Moreover, focusing on this prominent increase in Ki67 between days E14.5 and E15.5, which entirely coincides with the significant rise in the Nrf2 level (in the whole embryo and in the hindgut alone), we decided to correlate these two proteins. For each inspected timepoint, we observed a significant correlation; however, a very high coefficient of determination (r^2^) was denoted by E14.5 and E15.5—the days of active proliferation [3]. Some previous in vitro studies related to cancer have also suggested that Nrf2 activity influences Ki67 expression [24,25]. Given this striking increase in the Nrf2 level between E14.5 and E15.5 in the whole embryo, it would be worthy verifying whether this rise accounts for enhanced proliferation only at the intestinal level or at the organismal level. For instance, Nrf2 regulates the proliferation of enterocytes [15], hepatocytes [26,27], satellite cells [28], neuronal progenitor cells [29], and endothelial cells [16]. Thus, it is plausible that the regulation occurs at the organismal level and is not tissue specific.

In contrast, the lack of Nrf2 promotes the proliferation and differentiation of mouse small intestinal stem cells by the NF-κB signaling pathway [30]. Possibly, this is the factor governing the earlier proliferation of the Nrf2 tKO hindgut. Moreover, taking into account that day E14.5 is the starting point of intestinal remodeling, the formation of crypts, and proliferation [6], it is plausible that altered proliferation triggers abnormal microscopical features in Nrf2 tKO. Based on this, we suspect that Notch1 is not the main regulator responsible for these anomalies. However, it cannot be excluded that a significant drop in Notch1 expression at E18.5 in tKO mice accounts for other microscopical features such as the enlargement of goblet cells and irregularities.

In conclusion, our results show that the lack of transcriptionally active Nrf2 influences the hindgut morphology by modulating the expression of cell proliferation rather than the differentiation factors. It impacts the presence and localization of particular cell types in the intestines. These findings provide new insights into the role of Nrf2 in gut development and broaden our understanding of the biological functions of Nrf2.

## 4. Material and Methods

### 4.1. Animals

Animals of the following mouse strains were used: C57BL/6J mice with the functional Nrf2 (wild-type (WT)) or with the transcriptionally inactive form of Nrf2 (transcriptional knockout (tKO)). Mice were generated as described previously [17] and kindly provided by Prof. Antonio Cuadrado (Universidad Autonoma de Madrid, Spain). In these mice, a sequence coding for the carboxyl amino acid residues of Nrf2 (including DNA binding domain) was replaced by the LacZ gene. This results in the presence of the fusion protein Nrf2-LacZ, consisting of the remaining N-terminal 301 amino acids of Nrf2 being linked to β-gal. However, this fusion protein lost its DNA binding ability [16]. The animals were maintained under specific pathogen-free conditions in the individually ventilated cages (14/10 h light/dark cycle at a temperature of 22 ± 2 °C) and were provided with a standard diet and water ad libitum.

### 4.2. Embryo Preparation

Two-month-old male and female mice of the verified genotype were used for breeding. An impregnation was confirmed by the appearance of plug and fetuses at the gestation day (E)14.5, E15.5, E17.5, and E18.5 were harvested from the Nrf2 tKO and Nrf2 WT mice as previously described [31]. The uterus was removed from pregnant females, and fetuses were removed from the amniotic sack separately, washed in PBS, placed in 10% formalin for 24 h, and further incubated for 12 h in increased concentrations of sucrose (10%, 20%, and 30%). Finally, the fetuses were frozen in tissue freezing medium (O.C.T., Leica) and stored at −80 °C before further processing.

### 4.3. Genotyping PCR

The mice genotype was verified prior to the experiment by DNA analysis. The PCR reaction for Nrf2 was as follows: the thermocycler conditions were 95 °C for 3 min, followed by 35 cycles of 95 °C for 15 s, 62 °C for 15 s, and 72 °C for 15 s, with a final elongation at 72 °C for 1 min. Nrf2 primers—Nrf2 5′ TGGACGGGACTATTGAAGGCTG 3′; Nrf2-AS 5′ GCCGCCTTTTCAGTAGATGGAGG 3′; Lac Z 5′ GCGGATTGACCGTAATGGGATAGG 3′. For sex determination, DNA was isolated from the yolk sacs and analyzed for Rbm31 gene expression as previously described by [32] with some modifications. DNA was isolated using a Genomic Maxi AX Kit according to the manufacturer’s protocol (A&A Biotechnology). The PCR reaction was performed using a DNA solution. The thermocycler conditions were 94 °C for 2 min, followed by 35 cycles of 94 °C for 20 s, 55 °C for 20 s, and 72 °C for 2 s, with a final elongation at 72 °C for 5 min. Rbm31 primers—Forward: 5′ CACCTTAAGAACAAGCCAATACA 3′; Reverse 5′ GGCTTGTCCTGAAAACATTTGG 3′. PCR products were loaded onto a 2% (w/v) agarose gel containing ethidium bromide, electrophoresed in 1X TAE (40 mM Tris-acetate, 2 mM Na2EDTA) at 80 V/cm for 40 min and visualized on a UV transilluminator. The exemplary results of Rbm31 are presented in Figure S1.

### 4.4. Histological and Immunofluorescent Analysis

Immunofluorescent and immunohistochemical stainings were conducted on frozen 10 μm specimens. Hematoxylin and eosin (H&E) staining was performed using the modified method available on the IHCworld protocols website. Briefly, embryonic samples were stained with Mayer’s hematoxylin (Sigma-Aldrich) for 5 min, washed with tap water for 15 min, counterstained with eosin (Sigma-Aldrich) for 1 min, and dehydrated. Samples were analyzed under a light microscope (Nikon) with NIS elements BR software (Canon) at magnifications of 100× and 200×.

For the immunofluorescent staining, the samples were incubated in peroxidase blocking solution (Bio-Rad) for 10 min at room temperature (RT), washed once in washing buffer (WB, PBS without calcium and magnesium ions with 0.05% Tween-20), permeabilized in 0.5% Triton X-100 for 20 min at RT, and blocked in Mouse on Mouse (M.O.M.) blocking buffer (Vector Laboratories) with 1% bovine serum albumin (BSA; Lab Empire) in WB for 1 h at RT. After washing in WB, the samples were incubated overnight (4 °C) with mouse anti-Muc2 monoclonal IgG antibodies (dilution 1:400; Thermo Scientific), rabbit anti-ChrA IgG polyclonal antibodies (dilution 1:400; Novus Biologicals), prepared in 1% BSA in WB. The next day, the samples were washed and incubated with anti-rabbit or anti-mouse antibodies conjugated with Alexa Fluor 488 or Alexa Fluor 568 (dilution 1:1000; IgG H+L, Life Technologies) for 1 h at RT. Nuclei were counterstained with Hoechst 33,342 (1 μg/mL, Sigma-Aldrich) during the second washing. Negative controls are presented in Figure S2. The samples were analyzed under a fluorescent microscope (Nikon Eclipse TE 2000-U microscope fitted with a camera) at magnifications of 100×, 200×, and 400× or a meta laser scanning confocal microscope (LSM-880; Carl Zeiss) and analyzed using ImageJ software (Wayne-Rasband (NIH)).

For the immunohistochemical staining of Nrf2, Ki67, and Notch1, samples were incubated in peroxidase blocking solution (Bio-Rad) for 10 min at RT, washed once in PBS, incubated with Avidin blocking solution (Bio-Rad) for 10 min at RT, washed twice in PBS, blocked with Biotin blocking solution (Bio-Rad) for 10 min at RT, and then washed twice with PBS. Next, the samples were permeabilized in 0.2% Triton X100 for 10 min at RT and blocked in 10% goat serum (GS; Gibco) in PBS with 0.05% Tween-20 for Nrf2 and Notch1 or with M.O.M. blocking buffer (Vector Laboratories) in PBS with 0.05% Tween-20 for 1 h at RT. After washing in PBS, the samples were incubated overnight (4 °C) with mouse anti-Ki67 monoclonal IgG antibodies (dilution 1:500; Abcam), rabbit anti-Nrf2 IgG polyclonal antibodies (dilution 1:200; Proteintech), or rabbit anti-Notch1 IgG monoclonal antibodies (dilution 1:250; Cell Signaling) diluted in 3% GS in PBS with 0.05% Tween-20. The next day, the samples were washed and incubated with biotynylated goat anti-mouse/rabbit antibodies (ABC Kit, Abcam) for 10 min at RT, washed three times with PBS, and incubated with Streptavidin-HRP for 10 min at RT. After rinsing, the samples were incubated with DAB solution for 3 min and counterstained with Mayer’s hematoxylin for 5 s. Negative controls are presented in Figure S2. The samples were analyzed under a light microscope (Nikon) with NIS elements BR software (Canon) at magnifications of 200× and 400×. The quantification analysis of the protein content was conducted using the WEKA trainable segmentation 3D tool in Fiji [33]. Image processing is presented in Figure S3. The results are presented as % area and calculated for 5 to 10 images of the same sample.

### 4.5. Statistical Analysis

Data are presented as the mean ± SEM. The Student’s *t* test for a comparison of two groups or one-way ANOVA or two-way ANOVA, followed by Tukey’s post hoc test, was used for the comparison of more than two groups. The normality of data was confirmed by the Shapiro–Wilk test. Spearman’s test was applied to calculate the correlations. Grubbs’ test was used to detect the statistically significant outliers (*p* < 0.05), which were not included in the statistical analysis of the results (GraphPad Prism software). *p* < 0.05 was accepted as statistically significant.

## Data Availability

The data used to support the findings of this study are available from the corresponding author upon request.

## Supplementary Materials

The following supporting information can be downloaded at: https://www.mdpi.com/article/10.3390/ijms23116175/s1.
