# Supplementary material for: Nrf2 Transcriptional Activity Governs Intestine Development
Source: Int J Mol Sci. 2022 May 31;23(11):6175. doi: 10.3390/ijms23116175 (PMC9181470; doi:10.3390/ijms23116175)

Supplementary file to:

Nrf2 transcriptional activity governs intestine development

Aleksandra Kopacz, Damian Kloska, Dominika Klimczyk, Magdalena Kopec, Alicja Jozkowicz, Aleksandra Piechota-Polanczyk*

Jagiellonian University, Faculty of Biochemistry, Biophysics and Biotechnology, Department of Medical Biotechnology, Krakow, Poland

*Corresponding author

Department of Medical Biotechnology, Faculty of Biochemistry, Biophysics and Biotechnology, Jagiellonian University, Gronostajowa 7, 30-387 Krakow, Poland.
Email: aleksnadra.piechota-polanczyk@uj.edu.pl

Supplementary figures

Figure S1. Embryos genotyping for *Rbm31* gene. Numbers (consecutive embryos); M1 (mass marker 100-1000 kb); F- female; M- male.

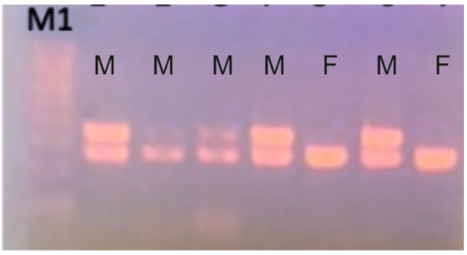

Figure S2. The negative controls for A) immunohistochemical and immunofluorescence stainings of B) 4-day-old pups and C) embryos. Representative images, magnification 400x, scale bar 30 µm for A and C. magnification 250x, scale bar 40 µm for B.

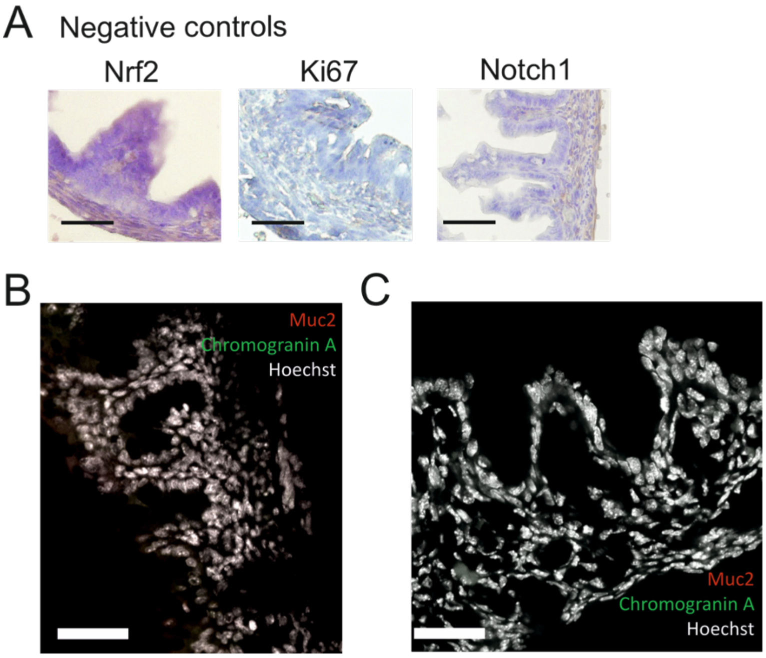

Figure S3. The workflow for the quantitative analysis of protein expression of IHC images.

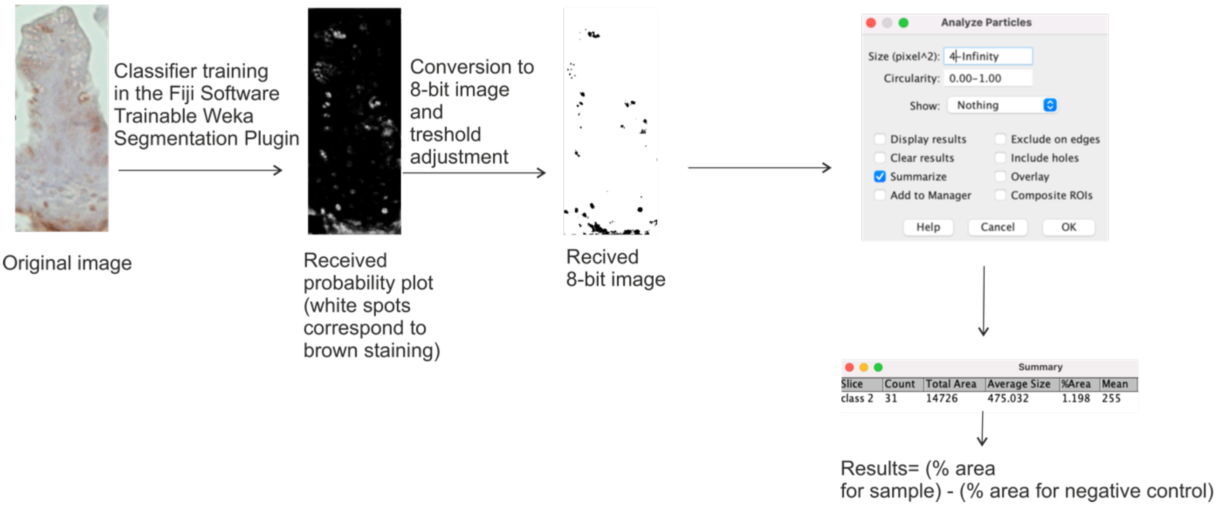

Supplement: Supplementary file 1 [file ijms-23-06175-s001.zip › ijms-1720690-supplementary.pdf]
